# MRI findings of an atypical testicular epidermoid cyst: A case report

**DOI:** 10.1097/MD.0000000000018818

**Published:** 2020-01-17

**Authors:** Renwei Liu, Aibo Li, Yixiang Jiang, Jiayin Ji, Shulin Yu, Nengxue Chen

**Affiliations:** a Urogenital System Group, Department of Radiology; b Department of Pathology; c Department of Ultrasonography, People's Hospital of Long Hua District, Shenzhen, China.

**Keywords:** benign intratesticular solid lesions, magnetic resonance images, neoplasms, testicular epidermoid cyst, testis

## Abstract

**Introduction:**

Typical testicular epidermoid cysts (TECs) manifestate as a target sign or onion skin sign on ultrasonography and magnetic resonance (MR) imaging. Clinicians are increasingly aware of the imaging characteristics of typical TECs, which allow accurate diagnosis and successful treatment while preserving the testicle, but atypical TECs are likely to be misdiagnosed as a malignant intratesticular neoplasm, leading to complete testicular resection.

**Patient concerns:**

A 26 year-old male patient complained of a painless enlargement of the left testicle that had been present for 1 month. The patient had no recent medical history of scrotal trauma or systemic infection.

**Diagnosis:**

A round 48 mm × 45 mm × 43 mm mass was seen inside the left testicle. T2-weighted images of the lesion showed a thin hypointense capsule. T1-weighted images of the lesion showed a hyperintense nodule on the cyst wall, which appeared hypointense on T2-weighted and SPAIR images. After Gd-DTPA injection, the lesion was not enhanced; however, the nodule was enhanced on THRIVE images. These manifestations were consistent with a benign intratesticular lesion, and MR imaging diagnosed atypical TEC, which was confirmed by pathology after surgery.

**Interventions:**

The patient was treated with organ-sparing surgery with testicular enucleation.

**Outcomes:**

The patient was re-examined with ultrasonography 3 months after surgery. The left residual testicular tissue appeared normal, and reproductive function was preserved.

**Conclusion:**

Urologists must be aware of the clinical and MR imaging characteristics of atypical TECs and the utility of preoperative MR imaging for the diagnosis of testicular lesions to ensure that organ-sparing surgery is performed rather than unnecessary orchiectomy.

## 1 Introduction

TECs are rare benign keratin-containing tumor-like lesions that occur in the testicles. TECs represent 1% to 2%^[1]^ of all testicular tumors, and usually occur in males aged 20 to 40 years.^[2]^ Clinically, TEC present as a painless enlargement of the testicle that manifests as a firm, well-defined palpable isolated mass or nodule in the testicular parenchyma on physical examination. Serum tumor markers, including AFP, β-HCG, and CEA are negative. Typical MR imaging findings of TECs, including “black ring, target, and onion skin signs,” have been reported in the literature,^[3]^ but atypical findings are rarely described. We present the case of a patient with a TECs confirmed by surgery and pathology that had atypical characteristics on preoperative ultrasonography and MR imaging.

## 2 Case presentation

**Figure 1:**
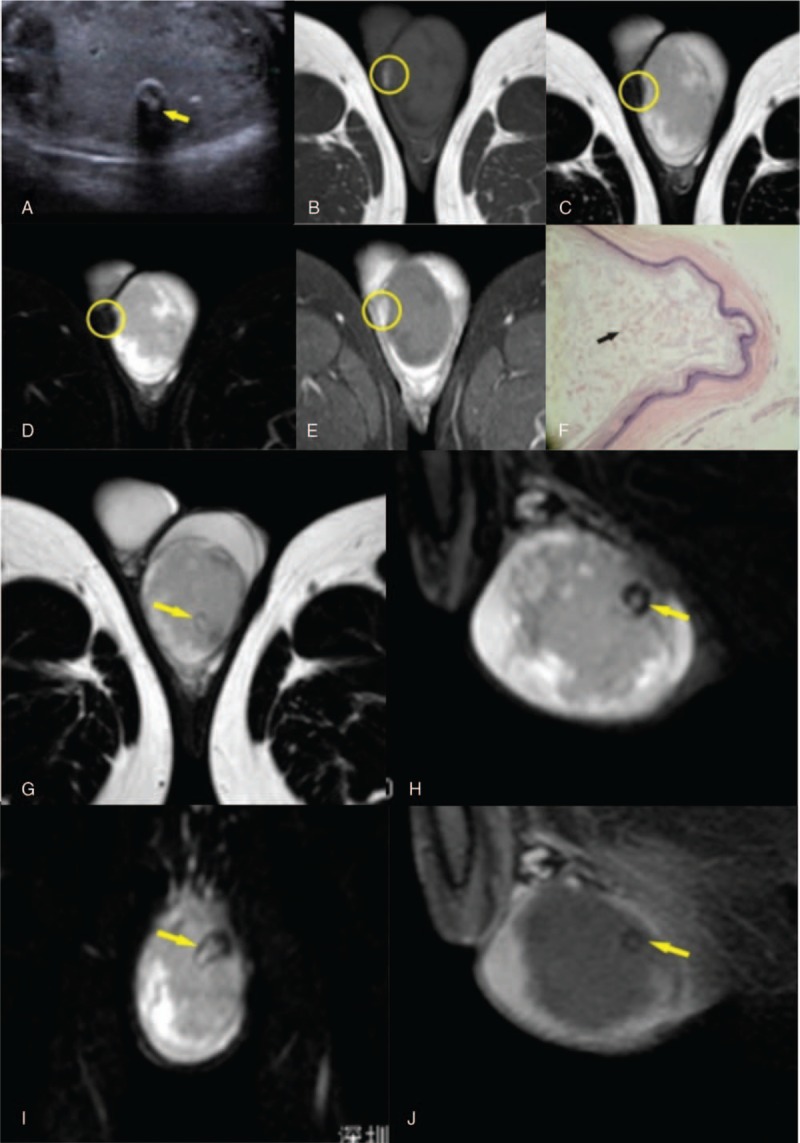
Twenty-six-year-old male who had a TECs with atypical characteristics on preoperative clinical examination and MR imaging. (A) Ultrasonography showing the lesion had a heterogeneous internal echotexture, with a central area of increased echogenicity accompanied by an acoustic shadow (yellow arrow). (B) T1-weighted images of the lesion showed a hyperintense nodule on the cyst wall (yellow circle). (C) T2-weighted images of the lesion showed a hypointense nodule on the cyst wall (yellow circle). (D) SPAIR images of the lesion showed a hypointense nodule on the cyst wall. (E) After Gd-DTPA injection, the nodule was enhanced on THRIVE images. (F) Pathology (HE × 20) showed a disorderly distribution of intracapsular keratides and no stratified structure (black arrow). (G–I) T2-weighted/SPAIR images displayed a small black ring sign. (J) After Gd-DTPA injection, the small black ring was not enhanced (yellow arrow) on THRIVE images.

A 26-year-old male patient presented to our institution with a painless enlargement of the left testicle that had been present for 1 month. Physical examination revealed a palpable mass within the testicle, routine blood and urine tests were normal, and serum AFP, β-HCG, and CEA were negative. Ultrasonography showed the diameter of the left testicle was increased but it had a normal shape and smooth surface. There was a 54 mm × 30 mm hyperechoic mass inside the testicle that had a regular shape and a distinct margin. The mass had a heterogeneous internal echotexture, with a central area of increased echogenicity accompanied by an acoustic shadow (Fig. 1A).

MR imaging was performed with a Philips Intera Achieva 1.5T Super conducting MR scanner and a phased array coil. Transverse T2-weighted images (repetition time [TR] 4500 ms, echo time [TE] 120 ms), transverse T1-weighted images (TR500 ms, TE 10 ms), transverse, coronal, and sagittal images with fat suppression using spectral attenuated inversion recovery (SPAIR) (TR3500 ms, TE 100 ms), and contrast-enhanced transverse, sagittal, and coronal images using a THRIVE sequence (TR 3.9 ms, TE 1.84 ms, thickness, 1.0 mm) were obtained with the following parameters: number of excitations (NEX) = 3, transverse images field of view (FOV) = 180 × 180, coronal and sagittal images FOV = 250 × 250, Gd-DTPA = 0.2 mmol/kg, flow rate = 2.0 mL/s.

MR imaging findings are shown in Figure 1B–E and G–J. The left testicle was enlarged. A round 48 mm × 45 mm × 43 mm mass was seen inside the testicle. The left testicle showed several hypointense areas on T1-weighted images, the signal intensity at the periphery was the same in both testicles, but the rest of the left testicle was hypointense on T2-weighted images.

T2-weighted images of the lesion showed a thin hypointense capsule. T1-weighted images of the lesion showed a hyperintense nodule on the cyst wall, which appeared hypointense on T2-weighted and SPAIR images. After Gd-DTPA injection, the lesion was not enhanced; however, the nodule was enhanced on THRIVE images. The lesion showed a small black ring on T2-weighted and SPAIR images, which was not enhanced on THRIVE images (Fig. 1J).

## 3 Discussion

The diagnosis of TECs is based on imaging. Typically, TECs appear as a “target sign“ or ”onion skin sign“ on ultrasonography and MR imaging depending on the amount and arrangement of keratin within the cyst. Langer^[4]^ proposed the “onion skin sign” as a specific marker of TEC on MR imaging based on findings from three patients. Liu^[3]^ noted that an “onion skin sign” and “black ring, target, and onion skin signs” are typical features of TEC on ultrasonography and MR imaging, respectively, based on findings from five patients. In the present case, ultrasonography and T1-weighted or T2-weighted images of the lesion revealed no ”target sign“ or ”onion skin sign“; therefore, the lesion was considered an atypical TECs. Although clinicians are increasingly aware of the imaging characteristics of typical TECs, which allow accurate diagnosis and successful treatment while preserving the testicle, atypical TECs are likely to be misdiagnosed as a testicular neoplasm, leading to complete testicular resection. The patient in the present case study was diagnosed as TECs by preoperative MR imaging, and organ-sparing surgery with testicular enucleation was performed, resulting in fertility preservation.

The characteristics of the atypical TECs described in the present case study, resemble those of two lesions included in the analysis of Cho et al, which showed heterogeneous mixed echogenicity with no alternating pattern on ultrasonography and MR imaging.^[5]^ However, the TECs in the present study had 3 additional features worth noting. First, T1-weighted images of the lesion showed a hyperintense nodule on the cyst wall, which was enhanced, but appeared hypointense on T2-weighted and SPAIR images. Pathology revealed that the nodule contained vascular and fibrous tissue. Second, T2-weighted images of the lesion showed a ”small black-ring sign,“ which may be attributed to the presence of a calcified shell.^[3]^ Third, the lesion did not demonstrate the typical “onion-ring” sign of alternating hypo- and hyperechogenic layers on T1-weighted or T2-weighted images. Histological analysis showed the cyst contained a disorderly accumulation of keratinized epithelium (Fig. 1F), described as ”bread crumbs"^[3]^ on gross pathology. The water and lipid content of materials within the cyst determined the signal intensity on T1-weighted and T2-weighted images.

The majority of intratesticular masses are malignant, and radical orchiectomy is the treatment of choice.^[6]^ Since atypical TECs are benign intratesticular lesions without a blood supply, differential diagnosis must first exclude testicular seminoma and nonseminomatous germ cell tumors, which have a blood supply. Atypical TECs should then be differentiated from the following three types of benign intratesticular lesions:

1. testicular cysts, which are usually detected incidentally, have signals characteristic of fluid on pulse sequences, and show no enhancement on contrast-enhanced MR imaging;
2. leydig cell hyperplasia, which is a rare, benign condition that is often bilateral and characterized by multiple small testicular nodules. Various conditions have been associated with leydig cell hyperplasia. On MR imaging, the multiple small nodules are hypointense on T2-weighted images, and there may be mild enhancement after administration of gadolinium contrast material;
3. hematoma, in which intracellular and extracellular methemoglobin within subacute blood is hyperintense on T1-weighted images.

T2-weighted images can demonstrate variable appearances. Chronic hematomas may show a hypointense rim on T2-weighted images secondary to hemosiderin deposition within macrophages. Preoperative MR imaging is very important for the diagnosis of various benign testicular lesions, for which radical orchiectomy is unnecessary.^[7]^ A more conservative approach, including follow-up, tumor enucleation or testis-sparing surgery may be justified.

According to the European Association of Urology guidelines, which were updated in 2011,^[8]^ the diagnosis of testicular cancer is based on clinical examination using imaging, serum tumor markers, and inguinal exploration and orchiectomy. There is the need for accurate evaluation of testicular lesions before surgery, and urologists must be aware of the utility of preoperative MR imaging for the diagnosis of TECs to ensure that organ-sparing surgery is performed rather than unnecessary orchiectomy.

## Author contributions

**Data curation:** Nengxue Chen.

**Formal analysis:** Aibo Li.

**Software:** Yixiang Jiang.

**Supervision:** Jiayin Ji.

**Validation:** Shulin Yu.

**Writing – original draft:** Renwei Liu.

**Writing – review & editing:** Renwei Liu.
